# Seropositivities against brucellosis, coxiellosis, and toxoplasmosis and associated factors in pregnant women with adverse pregnancy outcomes: A cross-sectional study

**DOI:** 10.1371/journal.pone.0216652

**Published:** 2019-05-09

**Authors:** Kan Kledmanee, Tippawan Liabsuetrakul, Somporn Sretrirutchai

**Affiliations:** 1 Epidemiology Unit, Faculty of Medicine, Prince of Songkla University, Songkhla, Thailand; 2 Department of Pathology, Faculty of Medicine, Prince of Songkla University, Songkhla, Thailand; INIA, SPAIN

## Abstract

**Background:**

Brucellosis, coxiellosis, and toxoplasmosis can be transmitted from infected ruminants to pregnant women and may induce adverse pregnancy outcomes; however, there are to date few studies. This study aimed to examine the seropositivities of immunoglobulin G (IgG) against those three pathogens among pregnant women with adverse pregnancy outcomes, and to explore the associated factors.

**Methods:**

A cross-sectional study was conducted in southern Thailand, where goat production is common. A total of 105 pregnant Thai women who had adverse pregnancy outcomes and serum samples collected at first antenatal care visit before their 28^th^ gestational week from June 2015 to June 2016 were included. The seropositivities of IgG anti-*Brucella abortus*, *Toxoplasma gondii*, and *Coxiella burnetii* antibodies were tested by using commercial enzyme-linked immunosorbent assay (ELISA) kits. Associated factors with seropositivity were analyzed using multiple logistic regression.

**Results:**

Most women were Muslim aged 20–34 years and 32.4% had a prior history of one or more adverse pregnancy outcomes. One-third of the women had been exposed to goats or raw goat products. Of the 105 serum samples, the seropositivity of anti-*T*. *gondii* IgG was highest (33/105, 31.4%), followed by anti-*C*. *burnetii* IgG (2/105, 1.9%), and anti-*B*. *abortus* IgG (1/105, 1.0%), respectively. None of the pregnant women were found to be co-seropositive for those three pathogens.

**Conclusions:**

One-third of women with adverse pregnancy outcomes showed positive antibodies for toxoplasmosis, coxiellosis and brucellosis. A dose-response relationship between seropositivity of anti-*T*. *gondii* IgG and age was noticed.

## Introduction

Miscarriage, stillbirth, premature birth, and low birth weight newborn are adverse pregnancy outcomes used as indicators for assessing the quality of maternal and child health services globally [1,2]. Among various other factors, these negative conditions can be caused by infection [3,4]. Many zoonotic pathogens including *Toxoplasma gondii*, *Chlamydia* sp., *Brucella* sp., and *Coxiella burnetii* can be transmitted from animals to pregnant women and lead to negative health consequences including adverse pregnancy outcomes [3–5]. One of the most important zoonotic disease reservoirs potentially impacting human health is domestic livestock, including cattle, camels, goats, and sheep. Small ruminants such as goats or sheep are reservoirs of many important zoonotic diseases, notably brucellosis caused by *Brucella* sp., coxiellosis caused by *C*. *burnetii*, and toxoplasmosis caused by *T*. *gondii* [6–8]. In endemic areas of these zoonotic pathogens, contact with infected animals and handling or ingesting raw animal products have been shown to be risk factors of these infections [9,10].

Several risk factors have been associated with human toxoplasmosis, particularly cat ownership and a history of raw meat consumption [11,12]. In pregnant women, infection with *T*. *gondii* can pose a serious risk for an adverse pregnancy outcome, including miscarriage, fetal anomaly, stillbirth, fetal growth restriction, and preterm birth [3,13]. Acute toxoplasmosis during pregnancy can also cause congenital toxoplasmosis [14]. A previous study found that women with a history of obstetric problems had a higher incidence of seropositivity for toxoplasmosis than women without any history of obstetric problems [15].

Brucellosis in humans is commonly caused by *B*. *melitensis* or *B*. *abortus* [16]. Milking animals and consumption of unpasteurized dairy products have been found to be risk factors for infecting human brucellosis [17,18]. Many studies have reported that brucellosis during pregnancy was a risk factor for obstetric complications, including congenital and neonatal infections [4,18,19]. A history of spontaneous abortion or intrauterine fetal death in pregnant women were associated with seropositivity for brucellosis [9].

Coxiellosis or Q fever in humans is primarily caused by inhalation of particles contaminated with birth secretions from an infected animal [20]. Occupational exposure to ruminants including goats is considered as a risk factor of human coxiellosis [21]. In pregnant women living in areas endemic for *C*. *burnetii*, coxiellosis during pregnancy has been associated with miscarriage and prematurity [22]. Additionally, another study reported that women with seropositivity for coxiellosis were more likely to have a previous or current obstetric complications [23].

Our study aimed to assess the seropositivities of immunoglobulin G anti-*Toxoplasma gondii* antibodies, immunoglobulin G anti-*Brucella abortus* antibodies and immunoglobulin G anti-*Coxiella burnetii* antibodies among pregnant women having adverse pregnancy outcomes in southern Thailand, and explore the associated factors with the seropositivities.

## Methods

### Study design and settings

A cross-sectional study was conducted in Songkhla Province in southern Thailand, where goat production is common among the Thai-Muslim communities and animal brucellosis is known to be endemic in the domestic goat population. [24,25]. The study carried out in Thepa, Na Thawee, Saba Yoi, and Chana districts of the province, where it has historically high rates of adverse pregnancy outcomes, including low birth weight infants ranging from 5.1–6.9% of total live births during the last decade [26,27]. The study settings were the primary care units in a district hospital or Health Promoting Hospital of four selected districts.

### Study participants

Pregnant Thai women aged 15–49 years coming for their first antenatal care (ANC) visit before their 28^th^ gestational week from June 2015 to June 2016 and ended with any adverse pregnancy outcomes, including miscarriage, stillbirth, premature birth, and low birth weight newborn were included. Miscarriage was defined as premature expulsion of an embryo or fetus at gestational age of 23 weeks or less or weighing less than 500 grams. Stillbirth was defined as birth of a fetus showing no signs of life. Premature birth was defined as a birth before the 37^th^ gestational week. Low birth weight was defined as a newborn weighing 2,500 grams or less. Those who had no serum samples taken at first ANC visit were excluded.

The formula of sample size calculation of this study considered the finite population that there was 150 women having adverse pregnancy outcomes in the study setting during enrollment period. According to the prevalence of seropositivity for zoonosis related with adverse pregnancy outcome among women in 22% [28] with precision of 5%, at least 96 women with adverse pregnancy outcomes were required.

### Data collection

A structured questionnaire was used to obtain the demographic characteristics of the participating women including age, religion, educational attainment, and occupation, as well as gathering obstetric information comprising gestational age at the first ANC, gravida, parity, and prior adverse pregnancy outcomes including miscarriage, stillbirth, premature birth, and low birth weight newborns. Pet ownership was defined as having any animals at home, and what types (if any). History of exposure with goat or its raw products before performing questionnaire considered as a risky history of brucellosis, coxiellosis and toxoplasmosis contraction were obtained. A history of exposure to goats was defined as having performed any activity in regard to raising goats. A history of physical contact with raw goat products was defined as having touched undercooked goat meat or milk through either occupational or daily household activities. A history of having ingested raw goat products was defined as having eaten uncooked goat meat or milk.

The serum samples of study women kept at -20 degrees Celsius in the Immunology and Virology Unit of Songklanagarind Hospital were tested for the presence of immunoglobulin G (IgG) for brucellosis and toxoplasmosis using Euroimmun anti-*Brucella abortus* and anti-*Toxoplasma gondii* enzyme-linked immunosorbent assay (ELISA) IgG (Lübeck, Germany), and coxiellosis using Vircell *Coxiella burnetii* ELISA IgG (Granada, Spain) kits. These commercial kits were run and interpreted according to the manufacturers’ protocols and recommendations. The detection of IgG, rather than IgM, was chosen to determine the immune status of these three pathogens in pregnant women.

### Data analysis

All data were double entered using EpiData version 3.1[29]. Statistical analysis was performed using R version 3.5.0 [30]. Distribution of adverse pregnancy outcomes and seroprevalence are descriptively presented in frequencies and percentages. The associations of demographic characteristics, obstetric information, type of animal at home, and adverse pregnancy outcomes were analyzed using multiple logistic regression. A two-sided *P* value of 0.05 was considered as statistically significant.

### Ethics considerations

This study was approved by the Research Ethics Committee of the Faculty of Medicine, Prince of Songkla University, Thailand (REC No. 58-061-15-1). The directors of the involved Provincial and District Public Health Offices gave their permission to conduct the research in each study setting. Written consent forms were obtained from all pregnant women after an oral explanation concerning the study was provided by the site research assistant. For pregnant women aged under 18 years, the consent of guardian was waived by the Research Ethics Committee.

## Results

The adverse pregnancy outcomes of the 105 included pregnant women were miscarriage (37.1%, 39/105), stillbirth (1.9%, 2/105), and premature birth or low birth weight newborn (61.0%, 64/105). Table 1 presents the demographic characteristics and related information of home animal and goat exposures. The mean age of the women was 27.9 ± 6 years. The majority were Muslim (91.4%). Half of them had graduated from secondary school and one-fifth of them were housewives. Seventy-five women (71.4%) had one or more home animals of which the most common was cats. One-third of them had experienced goat exposure with the majority of these involved in goat raising. The obstetric information of the women is presented in Table 2. Most women had attended their first ANC visit at gestational age of 12 weeks or less (86.7%) and two-thirds were multigravida (67.6%). Of the 105 women with current adverse pregnancy outcomes in our study, 32.4% had a prior history of one or more adverse pregnancy outcomes.

**Table 1:** Demographic characteristics and pet ownership information including animal at home and goat exposures.

Variables	Women (n = 105) n (%)
***Demographic characteristic***	
Age group, in years	
15–20	18 (17.1)
21–25	18 (17.1)
26–30	28 (26.7)
≥ 31	41 (39.1)
Religion	
Muslim	96 (91.4)
Buddhist or other	9 (8.6)
Education	
Primary school or lower	24 (22.9)
Secondary school	61 (58.1)
College/university or above	20 (19.0)
Occupation	
Housewife	23 (21.9)
Sales or office related	27 (25.7)
Laborer, agricultural or fishery worker	55 (52.4)
***Pet ownership and type of animal at home***	
No	30 (28.6)
Yes	75 (71.4)
Cat	53 (70.7)
Chickens or ducks	39 (52.0)
Caged bird	26 (34.7)
Goats or cattle	18 (24.0)
***Goat exposures***	
No	71 (67.6)
Yes	34 (32.4)
Performing any activity in goat raising	24 (70.6)
Ingestion of raw goat meat or milk	9 (26.5)
Physical contact with raw goat meat or milk	8 (23.5)

**Table 2:** Obstetric information.

Obstetric information	Women (n = 105) n (%)
Gestational age at first antenatal care visit, in weeks	
≤12	91 (86.7)
>12	14 (13.3)
Gravida	
Primi-gravida	34 (32.4)
Multi-gravida	71 (67.6)
Parity	
Nulliparity	36 (34.3)
Primiparity	34 (32.4)
Multiparity	35 (33.3)
Prior history of any adverse pregnancy outcomes	
No	71 (67.6)
Yes	34 (32.4)
Miscarriage	13 (38.3)
LBW	6 (17.7)
Premature birth	3 (8.8)
Stillbirth	0 (0.0)
Premature birth + LBW	7 (20.6)
Miscarriage + LBW	2 (5.9)
Stillbirth + LBW	1 (2.9)
Miscarriage + Premature birth + LBW	1 (2.9)
Stillbirth + Premature birth + LBW	1 (2.9)

LBW: low birth weight newborn

Thirty-six women with seropositive (34.3%) for antibodies against *B*. *abortus*, *C*. *burnetii*, or *T*. *gondii*. The seropositivity of IgG anti-*T*. *gondii* antibodies was highest (31.4%), followed by IgG anti-*C*. *burnetii* antibodies (1.9%), and IgG anti-*B*. *abortus* antibodies (1.0%) (Table 3). None of the women were found to be co-seropositive for antibodies against any of those three pathogens. Due to the small number of women with seropositivity for IgG anti-*B*. *abortus* and IgG anti-*C*. *burnetii*, the factors associated with being seropositive could be analyzed only for seropositivity for IgG anti-*T*. *gondii*. Women aged over 30 years or multiparous women were significantly more likely to show positive antibodies in univariate analysis, but there were no significant associations in the multiple logistic regression analysis (Table 4). The higher the woman’s age, the greater the odds ratios for positive antibodies in the dose-response relationship. Women with low education or who were a housewife were more likely to have higher odds of positive antibodies, but not to a significant level.

**Table 3:** Seropositivities for IgG anti-*Brucella abortus*, *Coxiella burnetii*, and *Toxoplasma gondii* antibodies.

Specific antibodies	Women (n = 105) n (%)
IgG anti-*Toxoplama gondii* antibodies	33 (31.4)
IgG anti-*Coxiella burnetii* antibodies	2 (1.9)
IgG anti-*Brucella abortus* antibodies	1 (1.0)

**Table 4:** Factors associated with seropositivity for IgG anti-*T*. *gondii* antibodies among pregnant women.

Factor	Seropositivity for IgG anti-*Toxoplasma gondii* antibodies		
	Crude OR (95% CI)	Adjusted OR (95% CI)	P value (LR-test)
*Demographic characteristic*			
Age group, in years (ref. = 15–20)			0.214
21–25	2.3 (0.4, 14.4)	2.6 (0.3, 20.8)	
26–30	4.4 (0.8, 23.4)	5.1 (0.7, 35.1)	
≥ 31	5.7 (1.2, 28.0)	8.1 (0.9, 72.4)	
Religion: Muslim vs Others	1.7 (0.3, 8.5)	2.8 (0.4, 17.4)	0.254
Education (ref. = College/university or above)			0.567
Primary school or lower	2.5 (0.7, 9.2)	2.6 (0.4, 14.7)	
Secondary school	1.2 (0.4, 3.7)	1.9 (0.4, 8.8)	
Occupation (ref. = Sales or office related)			0.173
Housewife	1.3 (0.4, 4.2)	3.3 (0.6, 17.3)	
Laborer, agricultural or fishery worker	1.1 (0.4, 2.9)	0.9 (0.2, 3.1)	
*Obstetric information*			
Gestational age at first ANC, in weeks: >12 vs ≤12	1.3 (0.4, 4.1)	1.3 (0.3, 5.1)	0.744
Parity (ref. = Nulliparity)			0.951
Primiparity	1.3 (0.4, 3.8)	0.9 (0.2, 3.4)	
Multiparity	3.0 (1.1, 8.3)	1.1 (0.2, 5.2)	
Prior history of any adverse pregnancy outcome: yes vs no	1.3 (0.5, 3.1)	0.6 (0.2, 2.0)	0.432
*Home animal*			
Cat: yes vs no	0.4 (0.2, 1.0)	0.4 (0.1, 1.1)	0.062
Chickens or ducks: yes vs no	0.6 (0.3, 1.6)	0.8 (0.3, 2.2)	0.601
Caged bird: yes vs no	0.6 (0.2, 1.6)	0.7 (0.2, 2.5)	0.559
Goat or cattle: yes vs no	0.4 (0.1, 1.4)	0.5 (0.1, 2.3)	0.326
*Goat exposures*			
Goat rearing, contact or ingestion of raw goat products: yes vs no	0.7 (0.3, 1.8)	1.2 (0.4, 3.6)	0.751

OR: odds ratio. CI: confidence interval of odds ratio. LR: likelihood ratio

## Discussion

The seropositivity of IgG anti-*T*. *gondii* antibodies was more commonly found among the study women than IgG anti-*C*. *burnetii* and IgG anti-*B*. *abortus* antibodies, but with only a non-significant dose-response relationship between seropositivity for IgG anti-*T*. *gondii* and the age of the women. There was also a potential relationship between IgG anti-*T*. *gondii* seropositivity with low educational attainment and being a housewife.

Toxoplasmosis seroprevalence among women of reproductive age has been found to vary widely depending on geographical regions in the world. Globally, toxoplasmosis seroprevalence among women was reported at up to 60% regardless of pregnancy status [31,32]. In Thailand, two studies reported the overall seroprevalences in non-pregnant women were 2.6% and 2.8% [33,34]; however, other studies reported a wide variation among pregnant Thai women ranging from 5.3% to 22.0% [28,35–38]. In our study, the toxoplasmosis seroprevalence was higher than in those previous reports from Thailand, which could be explained by noting that the pregnant women in our study were women who had had an adverse pregnancy outcome.

The seropositivity for IgG anti-*T*. *gondii* antibodies was shown to increase with age, similar to the findings of previous studies [12,39], which can be explained by noting that the chance of being exposed to sources of *T*. *gondii* will naturally increase with age. The association of education with seropositivity of toxoplasmosis in previous studies varied [11,40–42]. The study in Ethiopia showed women with low educational attainment had significantly higher odds of seropositivity, while in contrast, women with higher educational attainment in Burkina Faso were also more likely to be seropositive [39,41]. In our study, women with low educational attainment had higher odds of seropositivity of toxoplasmosis, but the difference was not statistically significant, which was similar to a study from Ethiopia [42]. This variation could be explained by other related factors such as residential area of the women [43] or environmental exposure to *T*. *gondii* contaminated animals through either daily activities or occupation [41]. Housewives had non-significantly higher odds of positive antibodies against toxoplasmosis than other occupation groups, similar to a previous study [11], which may be related to higher contact with raw animal products, especially meat, during food processing at home.

Very low seroprevalences of coxiellosis and brucellosis among pregnant women were found in our study (1.9% and 1.0%, respectively). This was a similar finding in animal that the seroprevalence of animal coxiellosis and brucellosis in Thailand were low as reported by previous studies (3.9% and 1.5%, respectively) [25,44]. In contrast, previous studies conducted among people having a livestock-related occupation in Thailand found that the seroprevalences of coxiellosis and brucellosis were 42.8% and 8.8%, respectively [44,45]. This may be due to the fact that the women participating in our study were not in animal-related occupations conferring a higher risk for the studied diseases. Thus, the exposure to raw animal products, including both meat and dairy products or occupational exposure to livestock, should be considered as the more important significant potential risk factor for coxiellosis and brucellosis.

This is the first study to measure the seropositivities of toxoplasmosis, coxiellosis and brucellosis in pregnant women who had adverse pregnancy outcomes in Thailand. There were some limitations to the study. First, the seropositivity tests for all three diseases were based on single serum samples collected at the first ANC visit, not paired serum samples, in detection of acute infection using IgG status. The seropositivities for toxoplasmosis, coxiellosis and brucellosis as found in our study could reflect a past exposure with the pathogens among the women. Second, the sample size was too small to explore the associations between seropositivities and potential risk factors as in the second objective because the sample size calculation was determined according to a prevalence study for the first objective. Finally, the study setting was an area where goat rearing is common, thus the findings are not generalizable to pregnant women in other settings, but our findings can still be useful as baseline information for antenatal care in settings where animal rearing, particularly goat rearing, is common, and also help to increase awareness of zoonotic diseases affecting maternal and newborn health.

In conclusion, one-third of women with adverse pregnancy outcomes showed positive antibodies for toxoplasmosis, while coxiellosis and brucellosis were less common. For toxoplasmosis seropositivity, a dose-response relationship with age was detected, and low educational attainment and being a housewife were found to be associated risk factors.

## Supporting information

S1 File Recording form used in data collection (English). (PDF)

S2 File Recording form used in data collection (Thai). (PDF)
