# Supplementary material for: Seropositivities against brucellosis, coxiellosis, and toxoplasmosis and associated factors in pregnant women with adverse pregnancy outcomes: A cross-sectional study
Source: PLoS One. 2019 May 9;14(5):e0216652. doi: 10.1371/journal.pone.0216652 (PMC6508713; doi:10.1371/journal.pone.0216652)
Supplement: S1 File — (PDF) [file pone.0216652.s001.pdf]

Recording form

Project: Seropositivities against brucellosis, coxiellosis, and toxoplasmosis and associated factors in pregnant women with adverse pregnancy outcomes

Date of data collection____/____/____

Instruction: Please check the appropriate box and fill in the information of pregnant woman

Section 1: General information **wid [ ] [ ] [ ]**

1. Date of birth (dd/mm/yy) ________/________/________ Age________years age [ ][ ]

2. Religion ☐ 1. Buddhist ☐ 2. Muslim ☐ 3. Christianity ☐ 4. Others, please specify____________ relig [ ]

3. Educational attainment ☐ 1. Primary school or lower ☐ 2. Secondary school ☐ 3. College or university edu [ ]

4. Occupation ☐ 1. No/housewife ☐ 2. Agricultural farmer ☐ 3. Livestock farmer ☐ 4.Fisherman occ [ ] [ ]
☐ 5. Laborer ☐ 6. Salesperson ☐ 7. Private officer
☐ 8. Others, please specify______________________________

5. Home animal ☐ 1. No ☐ 2. Cat ☐ 3. Dog ☐ 4. Caged bird ☐ 5. Chicken pet[ ] [ ]
☐ 6. Duck ☐ 7. Goat ☐ 8. Others, please specify____________________

Section 2: Obstetric information

6. Gestational age at present ________weeks ga [ ] [ ]

7. Gestational age at date of blood collection ____________weeks bldga [ ] [ ]

8. Gravida ______________________________ grav [ ] [ ]

9. Parity ______________________________ par [ ] [ ]

10. Have any of following history?

10.1 Miscarriage ☐ 1. No ☐ 2. Yes ______ times abhis [ ]
10.2 Stillbirth ☐ 1. No ☐ 2. Yes ______ times sbhis [ ]
10.3 Preterm birth ☐ 1. No ☐ 2. Yes ______ times pthis [ ]
10.4 Low birth weight newborn (<2,500 grams) ☐ 1. No ☐ 2. Yes ______ times lbwhis [ ]

11. Plan of delivery, please specify the hospital name______________________________ brthhosp [ ]

Section 3: History of goat and raw goat products exposure

12. Performing any activity in goat raising ☐ 1. Never ☐ 2. Ever gtrear [ ]

13. Ingestion of raw goat meat or milk ☐ 1. Never ☐ 2. Ever csmrw [ ]

14. Physical contact with raw goat meat or milk ☐ 1. Never ☐ 2. Ever ctrw [ ]

Section 4: Follow-up pregnancy outcome

15. Pregnancy outcome [ ] 1. Miscarriage at GA ______ weeks [ ] 2. Stillbirth at GA ______ weeks pregoc [ ]
[ ] 3. Preterm at GA ______ weeks [ ] 4. LBW at GA ______ weeks pregocga [ ] [ ]

Section 5: Serological results

Specimen ID ______________________________LAB ID ______________________________

16. ELISA ○ Toxoplasmosis ☐ 1. Negative ☐ 2. Positive (note:____________________) toxo [ ]
○ Brucellosis ☐ 1. Negative ☐ 2. Positive (note:____________________) bru [ ]
○ Coxiellosis ☐ 1. Negative ☐ 2. Positive (note:____________________) cox [ ]

hospid [ ] [ ] [ ] wid [ ] [ ] [ ]
dd/mm/yy [ ] [ ] / [ ] [ ] / [ ] [ ]
