# Supplementary material for: Seropositivities against brucellosis, coxiellosis, and toxoplasmosis and associated factors in pregnant women with adverse pregnancy outcomes: A cross-sectional study
Source: PLoS One. 2019 May 9;14(5):e0216652. doi: 10.1371/journal.pone.0216652 (PMC6508713; doi:10.1371/journal.pone.0216652)
Supplement: S2 File — (PDF) [file pone.0216652.s002.pdf]

แบบบันทึกข้อมูล

โครงการวิจัยเรื่อง ความชุกและปัจจัยที่เกี่ยวข้องต่อการพบผลบวกทางซีรัมต่อโรคบรูเซลโลซิส โรคไข้คิว และโรคทอกโซพลาสโมซิส ในหญิงตั้งครรภ์ที่มีผลลัพธ์อันไม่พึงประสงค์ของการตั้งครรภ์

วันที่เก็บข้อมูล______/______/________

คำชี้แจง: กรุณาทำเครื่องหมาย ✓ ลงใน ☐ หน้าข้อความและเติมข้อมูลที่ตรงกับหรือใกล้เคียงกับความเป็นจริง

ตอนที่ 1: ข้อมูลทั่วไปของหญิงตั้งครรภ์ wid [ ] [ ] [ ]

1. เกิดวันที่______________เดือน_____________________พ.ศ.______________ อายุ______________ปี age [ ][ ]

2. ศาสนา ☐ 1.พุทธ ☐ 2.อิสลาม ☐ 3. คริสต์ ☐ 4.อื่นๆ โปรดระบุ______________________________ relig [ ]

3. ระดับการศึกษาสูงสุด ☐ 1.ประถมศึกษาหรือไม่ได้รับการศึกษา ☐ 2.มัธยมศึกษา ☐ 3.อนุปริญญาหรือปริญญาตรีขึ้นไป edu [ ]

4. อาชีพปัจจุบัน ☐ 1.ไม่มีอาชีพ/อยู่บ้านเฉยๆ ☐ 2.เกษตรกรทำสวน ทำไร่ ☐ 3.เลี้ยงสัตว์ ☐ 4.ประมง occ [ ] [ ]
☐ 5.รับจ้าง ☐ 6.ค้าขาย ☐ 7.พนักงานเอกชน
☐ 8.อื่นๆ โปรดระบุ____

5. สัตว์เลี้ยงในบ้าน ☐ 1.ไม่มี ☐ 2.แมว ☐ 3.สุนัข ☐ 4.นก ☐ 5.ไก่ pet[ ] [ ]
☐ 6.เป็ด ☐ 7.แพะ ☐ 8. อื่นๆ โปรดระบุชนิด ______________________

ตอนที่ 2: ข้อมูลเกี่ยวกับการตั้งครรภ์ของหญิงตั้งครรภ์

6. อายุครรภ์ปัจจุบัน ___________สัปดาห์ ga [ ] [ ]

7. อายุครรภ์ที่ได้รับการเจาะเลือดตรวจ ________________สัปดาห์ bldga [ ] [ ]

8. จำนวนครั้งที่เคยตั้งครรภ์ (รวมครรภ์ปัจจุบันด้วย) ____ ครั้ง grav [ ] [ ]

9. จำนวนครั้งที่เคยคลอดบุตร ____ ครั้ง par [ ] [ ]

10. เคยมีประวัติการตั้งครรภ์/คลอดที่ผ่านมาต่อไปนี้หรือไม่

10.1 แท้ง ☐ 1.ไม่มี ☐ 2.มี จำนวน _________ ครั้ง abhis [ ]

10.2 ทารกเสียชีวิตก่อนคลอด ☐ 1.ไม่มี ☐ 2.มี จำนวน _________ ครั้ง sbhis [ ]

10.3 คลอดก่อนกำหนด ☐ 1.ไม่มี ☐ 2.มี จำนวน _________ ครั้ง pthis [ ]

10.4 ทารกน้ำหนักน้อยกว่า 2,500 กรัม ☐ 1.ไม่มี ☐ 2.มี จำนวน _________ ครั้ง lbwhis [ ]

11. ท่านวางแผนจะคลอดที่ใด โปรดระบุชื่อสถานพยาบาลและอำเภอ____ brthhosp [ ]

ตอนที่ 3: ประวัติการสัมผัสแพะและผลิตภัณฑ์ดิบจากแพะ

12. เลี้ยงแพะหรือทำกิจกรรมที่เกี่ยวข้องกับการเลี้ยงแพะ ☐ 1.ไม่เคย ☐ 2.เคย gtrear [ ]

13. รับประทานเนื้อแพะดิบหรือนมแพะดิบ ☐ 1.ไม่เคย ☐ 2.เคย csmrw [ ]

14. สัมผัสเนื้อแพะดิบหรือนมแพะดิบ ☐ 1.ไม่เคย ☐ 2.เคย ctrw [ ]

ตอนที่ 4: ผลการติดตามผลลัพธ์การตั้งครรภ์ของหญิงตั้งครรภ์

15. สรุปผลลัพธ์ [ ] 1. Miscarriage ที่อายุครรภ์ _______ สัปดาห์ [ ] 2. Stillbirth ที่อายุครรภ์ ________ สัปดาห์ pregoc [ ]
[ ] 3. Preterm ที่อายุครรภ์ _______ สัปดาห์ [ ] 4. LBW ที่อายุครรภ์ _______ สัปดาห์ pregocga [ ] [ ]

ตอนที่ 5: ผลตรวจทางซีรัมวิทยา

Specimen ID ____LAB ID ____

16. ELISA
○ Toxoplasmosis ☐ 1. Negative ☐ 2. Positive (note:____) toxo [ ]
○ Brucellosis ☐ 1. Negative ☐ 2. Positive (note:____) bru [ ]
○ Coxiellosis ☐ 1. Negative ☐ 2. Positive (note:____) cox [ ]

hospid [ ] [ ] [ ] wid [ ] [ ] [ ]
dd/mm/yy [ ] [ ] / [ ] [ ] / [ ] [ ]
